# Patient Awareness of Reported Adverse Effects Associated with Proton Pump Inhibitors in a Medically Underserved Community

**DOI:** 10.3390/healthcare8040499

**Published:** 2020-11-19

**Authors:** Brian White, Matthew Drew, John Gaughan, Sangita Phadtare

**Affiliations:** 1 Department of Biomedical Sciences, Cooper Medical School of Rowan University, 401 S Broadway, Camden, NJ 08103, USA; whiteb64@rowan.edu; 2 Department of Gastroenterology, Cooper University Health Care, 1 Cooper Plaza, Camden, NJ 08103, USA; mdrew@agcosprings.com; 3 Associates in Gastroenterology, 2940 North Circle Drive, Colorado Springs, CO 80909, USA; 4 Department of Biostatistics, Cooper University Health Care, 1 Cooper Plaza, Camden, NJ 08103, USA; gaughan@rowan.edu

**Keywords:** proton pump inhibitors, patient knowledge, adverse effects, medically underserved, patient education, biopsychosocial

## Abstract

Reports of adverse effects associated with proton pump inhibitors (PPIs) are concerning because of high usage and over-the-counter availability. We sought to determine the awareness of PPI adverse effects among our patient population, which is medically underserved, low-income, and racially diverse. A 21-item survey was administered to gastroenterology-clinic outpatients. It collected information about age, gender, education, race, specialty of the prescriber, specific PPI, indication, knowledge of dose, adherence, duration of use and awareness of any risks. Medical records were reviewed to verify survey responses pertaining to indication, dosing, and adherence. A vast majority (96%) of 101 participants were not aware of PPI adverse effects. In total, 63% of the patients completed a high school education or less, which was associated with a higher risk of long-term PPI use than completion of at least an undergraduate degree (*p* = 0.05). In contrast to other studies, the shockingly low patient awareness about PPI adverse effects in our patient population is particularly concerning, especially as it is tied to their demographic attributes. This may lead to long-term and high-dose PPI use. Our study highlights the need for effective provider-driven education regarding medication risks, especially in the communities with significant health disparities.

## 1. Introduction

Proton pump inhibitors (PPIs) are among the most widely prescribed medications in the world, and the increasing availability of over-the-counter (OTC) PPIs has contributed to even greater use [1,2]. Their use has increased significantly over the past few years. In the United States, nearly one in five older adults is estimated to take a PPI, many on a long-term basis [3,4,5]. PPIs have proven indications for multiple acid-related conditions, including gastroesophageal reflux disease (GERD), erosive esophagitis, Barrett’s esophagus, peptic ulcer disease (PUD), *Helicobacter pylori* eradication, and non-steroidal anti-inflammatory (NSAID)-associated ulcer prophylaxis in high-risk patients [6,7]. Although PPIs are generally well tolerated, a growing number of studies have reported associations with adverse effects over the last decade [6]. Associations of PPI use with *Clostridium difficile* infection, pneumonia, kidney disease, dementia, decreased bone mineral density, micronutrient deficiencies, and interactions with antiplatelet agents have been reported [2,6,8,9,10,11]. Notably, at the time of submission of this manuscript, a survey-based study was published which suggested an increased risk of COVID-19 test positivity among patients taking PPIs [12]. Although the quality of evidence for these associations is variable, these adverse effects are nonetheless concerning, particularly in the light of the many studies that have identified rates of inappropriate short and long-term PPI use well over 50% [5,13,14,15,16]. Consequently, perception of risks associated with PPIs has prompted many providers to alter their prescription practices [17].

While the potential adverse effects of PPIs use have garnered increasing attention among healthcare providers, patient perceptions of these associated risks are less understood [17]. Substantial differences have been observed in the perception of medication toxicity in general between healthcare providers and patients, although one survey of hospitalized patients suggested that they similarly regard PPIs as relatively safe medications [18,19]. Other studies indicate that up to 60% of patients taking PPIs worry about potential adverse effects, and most want to minimize their exposure to PPIs by taking the lowest effective dose or taking them only when needed [20,21,22]. Nevertheless, it is not unclear to what extent patients are aware of these reported adverse effects, nor is it well understood whether risk perception has affected prescription behavior.

Camden County is the most racially diverse of the four counties in southern New Jersey, with the lowest median income and highest poverty and unemployment rates [23]. It also has the lowest educational attainment and the greatest socioeconomic disparities among ethnic minority groups. In total, 37.4% of the population in Camden City, where our hospital is located, lives below the poverty line, and the median household income is USD 26,105 [23]. Almost one quarter (23.5%) of adults over 25 years old have not completed high school. Our hospital system has a community-based health focus and is committed to caring for a medically underserved, *low-income,* and minority patient *population*. These biopsychosocial disparities increase the risks of improper prescription medication usage and associated complications. As part of our continuous patient care quality improvement efforts, we sought to assess the awareness of reported adverse effects associated with PPIs and characteristics of use among patients in communities with low socioeconomic status and low educational attainment. In this study, we describe the outcomes of a survey conducted among outpatients at our community-based tertiary medical center. We aim to use outcomes from this study to incorporate provider-driven education in our practice to enhance informed decision-making and improve patient care.

## 2. Materials and Methods

This was a prospective study performed over two years (March 2016–February 2018). The study received ethical approval from the Cooper University Hospital Institutional Review Board (IRB) (Reference number 17-007). Informed consent was obtained from all individual participants included in the study. Patient anonymity was preserved throughout the study. Judicious inclusion and exclusion criteria were employed to minimize sampling bias. Patients receiving care at the Cooper Digestive Health Institute and who were either currently taking a prescribed PPI or took a prescribed PPI within the past year were eligible for inclusion. Patients who were (i) not a Cooper Digestive Health Institute patient, (ii) not taking a proton pump inhibitor currently nor in the past year, or (iii) unable to consent were excluded. Included patients were asked to complete paper surveys after giving consent for participation. There are no data in our patient community to indicate how many patients are aware of the side effects of PPIs, so we recruited as many eligible patients as possible over the two-year study period. No particular preference was given to demographics or any other patient characteristics while recruiting. We recruited all those who were willing to participate in the study during this study. We recruited all the eligible patients who were willing to participate in the two-year time period mentioned above. This helped to avoid a potential bias in recruiting. A total of 101 patients gave their consent to participate and all of these completed the survey. Before we enrolled patients in the study, we ensured that the survey questions were easy to follow by administering the survey to ten control subjects. The two-page survey included only multiple choice questions. The participating patients had to only circle in the choices given, which made it easier and time efficient for them to take the survey. The survey is included in the Supplementary Materials to this article.

The survey was divided in three parts. The first part collected demographic information, such as age, gender, ethnicity/race and education level. It also included questions such as (i) Doctor who prescribed PPI; (ii) Which PPI do/did you take?; (iii) Do you know your dose of PPI?; (iv) How long you were or are on PPI?; (v) How often did your doctor recommend you take PPI?; (vi) How often do/did you take PPI?; (vii) Reason to take PPI; (ix) Are you aware of side effects of PPIs? Patients reporting awareness of risks were inquired about their awareness of specific associations with *C. difficile* infection, pneumonia, kidney disease, and dementia, and were prompted to list any others not listed in the survey. As mentioned above, to evaluate adherence, patients were asked to select the answer choice corresponding to the frequency with which they took their PPI (i.e., one time every day, two times every day, more than two times every day, etc.) and these responses were compared with the prescription details documented in the patient’s medical record. Adherence was considered to be consistency between the patient-reported frequency of use and the documented prescription frequency.

If the patients were not aware of the adverse effect of the PPI, then they were not required to complete parts 2 and 3 of the survey. Patients who were aware of the side effects of PPI were asked: (i) Choose all specific side effects of PPIs of which you are aware; (ii) How did you hear about PPI side effects?; (iii) Because of side effects, have you changed the dose, frequency, or stopped PPI?;

Patients who changed the dose, frequency, or stopped PPI because of the side effects continued taking part 3 of the survey with questions such as (i) Have you stopped the medicine completely?; (ii) Do you take it less often?; (iii) Did you switch to lower dose?; (iv) If you stopped completely, did you switch to a different medication?; (v) Did you discuss any of these changes with your doctor?

Medical records were reviewed to verify survey responses pertaining to indication, dosing, and adherence. Statistical analysis was performed using the statistical software package SAS v9.4 (SAS Institute, Cary, NC, USA). Data were cross-tabulated by age, gender, race, and education level for analysis. Fisher’s exact test was used to compare results across the different strata. All data are reported as percentages. *p* values ≤ 0.05 were considered statistically significant.

## 3. Results

### 3.1. Demographics

Among the 101 participating patients, 62% were female. In total, 69% were Caucasian, and 20% and 10% were African American, and Hispanic, respectively. More than half of the patients were aged 51–70 years. In total, 63% of patients completed high school education or less (Table 1).

### 3.2. PPI Prescription Characteristics

The majority (69%) of patients had PPI prescriptions from a gastroenterologist, while 25% had prescriptions from a primary care physician. A small fraction of patients had PPI prescriptions from an Otolaryngologist (2%) or another type of provider (4%). Omeprazole constituted the majority of PPI prescriptions (52%), followed by pantoprazole (28%) and esomeprazole (15%). Lansoprazole (3%), dexlansoprazole (2%), and rabeprazole (1%) were not commonly prescribed. In total, 64% of prescriptions were indicated for GERD. Other indications included peptic ulcer disease (5%), Barrett’s esophagus (14%), nonsteroidal anti-inflammatory drug (NSAID)-associated ulcer prophylaxis (2%) and dyspepsia (10%) (Table 2).

### 3.3. Duration of PPI Use, Knowledge of Dose, and Adherence

Long-term PPI use was very prevalent in our sample. In total, 65% of patients were taking a PPI for 1 year or more, including 10% who had been taking it for more than 10 years (Table 2). Patients with a high school education or less were more likely to be taking a PPI for 1 year or more compared to those with an undergraduate degree or higher (72% *versus* 51%; *p =* 0.05; Table 3).

No significant differences in duration of PPI use were identified among different racial groups or genders (Table 4 and Table 5).

There was a trend towards a higher rate of PPI use for 1 year or more among males, but this did not meet statistical significance (74% males versus 59% females; *p =* 0.14; Table 5). In total, 57% of patients knew their prescribed dose; this rate was consistent across different age groups, genders, and levels of education (Table 2, Table 3, Table 4 and Table 5). Knowledge of prescribed dose was higher among Caucasians than among African Americans and Hispanics, combined (70% versus 29%; *p* <0.01; Table 4). In total, 22% of patients did not adhere to their prescribed regimen (Table 2). No significant differences were identified in rates of adherence among different age groups, racial groups, genders, or levels of education (Table 3, Table 4 and Table 5). There was a trend towards a higher rate of adherence among Caucasians compared to African Americans and Hispanics, combined, but this did not meet statistical significance (84% versus 68%; *p =* 0.11; Table 4).

### 3.4. Patient Awareness of Adverse Effects Associated with PPIs

Patient awareness of adverse effects associated with PPIs was very low in our overall population and across all subgroups. Four patients reported awareness of risks associated with PPIs. One reported awareness of an association with *C. difficile* infection, one of an association with kidney disease and dementia, one of an association with dementia alone, and one of an association with bone disease. Sources of information among the patients who reported awareness of risks were varied. One patient reported learning of adverse effects from the news and internet, one from the internet alone, and two from the manufacturer’s package insert. None of the four patients altered prescription habits based on their awareness of reported adverse effects. The sample was not large enough to detect significant differences in awareness of adverse effects when stratified by the different demographic variables, including level of education or prescribing characteristics, such as indication. All four patients reporting awareness of risks associated with PPIs were Caucasian, and three of these were female. Among the four, one did not complete a high school education, one completed high school, and two completed undergraduate education.

### 3.5. PPI Discontinuation

Very few patients in our population had recently discontinued their PPI. Four patients reported recently discontinuing their PPI, each for a different reason. One discontinued due to deprescription by their provider, one due to lack of efficacy, another due to cost, and the last due to decision to switch to a different therapy. Notably, none of these patients discontinued their PPI due to concerns about potential adverse effects.

## 4. Discussion

Patient-centered care (PCC) is an evidence-based approach that focuses on defining outcomes that are meaningful and valuable to the individual patient [24]. PCC emphasizes a patient-provider partnership that promotes patient knowledge and engagement, among others [24]. Widespread efforts are being made to implement PCC across U.S. healthcare systems, but lack of awareness regarding medication risks remains a barrier to patients becoming more active participants in their care. Patients increasingly depend on their provider to inform them of all potential risks associated with their medications, and neglecting to share such information may adversely affect the doctor–patient relationship [18,25].

Our goal was to assess patient awareness of reported adverse effects associated with PPIs. Nearly all (96%) of our surveyed patients reported no awareness of PPI adverse effects, and this finding suggests that current PPI patient education strategies among our study population do not emphasize associations with adverse effects. This conclusion is consistent with that from a recent study of Veterans receiving PPIs for GERD [26]. Although patients in this study reported that their GERD-related care was well-organized and supportive for decision-making, they noted gaps in PPI risk communication using validated patient experience measures [26]. This knowledge gap regarding PPI adverse effects is similarly evident in our study.

We recruited all the patients who were willing to participate in the study. GERD constituted the majority of indications for PPI prescriptions in our sample, which is consistent with other studies [5,27]. It is not clear whether PPI prescription for GERD is associated with a lower awareness of adverse effects when compared to prescriptions for other conditions, such as peptic ulcer disease, as our study was not powered to make such comparisons.

Our study included a high proportion of females (62%), but none of the outcomes varied significantly by gender, and this factor is unlikely to account for the observed low rate of awareness of PPI adverse effects. Notably, the ethno-racial distributions of our sample track closely with those of the background population at the time of study, with the exceptions of a higher proportion of Caucasians (69% vs. 57%) and slightly lower proportion of Hispanic individuals (10% vs. 18%) [23]. Our sample thus offers a reasonable representation of the studied population’s awareness of PPI adverse effects.

Our study further describes the lack of risk perception regarding PPIs among a population characterized by a high poverty rate and limited educational attainment [28]. Of note, 63% of the patients in our study completed a high school education or less. The low awareness of reported adverse effects associated with PPIs in this population is particularly relevant because of the extent of long-term and high-dose PPI use observed among patients with low educational level and low socioeconomic status [29,30,31]. Indeed, 65% of our participants were using a PPI for 1 year or more. This substantial rate of long-term use may be partially attributable to the severity of our patients’ conditions, which necessitated evaluation by a gastroenterologist and prescription of PPIs (69%). Specifically, completion of high school or less was associated with a higher risk of long-term PPI use than completion of an undergraduate degree or higher (72% versus 51%; *p =* 0.05). A conclusion cannot be drawn regarding if prescriptions by gastroenterologists contributed to the low rate of awareness of adverse effects. It is possible that these patients have more confidence in their specialist doctor’s prescriptions and regard them as safe for this reason. However, the other side of the coin is that this may lead to higher misuse of PPIs, especially as these are available over the counter. Patients with lower education level are likely to use PPIs and other OTC medications even after the treatment is over having confidence in the medications as safe as these were prescribed by a specialist before. Our findings suggest that low awareness of reported adverse effects associated with PPIs among patients in similar populations may have even greater impact due to the higher prevalence of long-term use especially in the background of lower education level.

The socioeconomic and educational characteristics of our population may account for differences in patient perceptions of risks associated with PPIs from studies published by Ghosh et al. and Kurlander et al. [27,32]. In total, 45% of surveyed patients from the Ghosh study reported knowledge of adverse effects associated with PPIs, but this rate was significantly lower among patients who did not complete high school [31]. In total, 20% of patients were able to write at least one potential adverse effect associated with PPIs in the Kurlander study, which surveyed a national population that included a smaller proportion of non-white patients (9%) with distinct educational characteristics [32]. Interestingly, 38% of PPI users from the Ghosh study changed their medication behavior based on concerns about adverse effects, and a similar number from the Kurlander study reported attempts to stop their PPI, most without recommendation from their provider [27,32]. We were not able to detect whether patients changed their medication behavior based on concerns about adverse effects, due to the small number of patients in this subset. Ghosh et al. reported that patients were most often acquiring information about adverse effects from non-physician sources [27]. Although all four patients in our study reporting awareness of PPI adverse effects learned of them from non-physician sources, our sample is not large enough to make firm conclusions about the predominant source of medication safety information in our study population.

Patient awareness of reported adverse effects associated with PPIs may have implications for inappropriate PPI prescription. Rates of inappropriate PPI prescription range from 27 to 81% in hospitalized patients and 36 to 63% in patients in primary care settings [14]. Multiple strategies have been described to identify PPI overuse and reduce rates of inappropriate prescription, including provider education initiatives and guideline-driven medication review by independent pharmacists and gastroenterologists [33,34,35]. While some studies have reported success with such strategies, the role of patient education in reducing inappropriate PPI use has not been fully elucidated. In one study of an intervention to reduce PPI prescription in primary care, surveyed patients rated communication about the reasons for reducing PPI use as highly important [36]. Moreover, they placed an emphasis on being involved in the decision to reduce their PPI use [36]. For these reasons, it is likely that stronger risk communication would improve provider-driven efforts to reduce unnecessary PPI use. Indeed, a recent quality improvement study which included a patient education component showed ~30% reduction in inappropriate PPI prescriptions over 12 months [34]. Additionally, there is encouraging evidence that patient-directed strategies can reduce inappropriate prescriptions. Of note, a randomized trial demonstrated that pamphlets prompting patients to approach their provider to periodically re-evaluate their need for long-term PPI use significantly reduced the volume of long-term prescriptions [37].

Greater awareness of the potential adverse effects associated with PPIs may limit the inappropriate use of OTC PPIs. The participating subjects in this study were prescribed PPIs mostly by gastroenterologists. It is thus expected that they would know about the adverse effects of PPI. However, as seen from our data, that is not the case. This raises serious concern about the lack of awareness in the general population using over the counter (OTC) PPIs. The population using OTC PPIs undoubtedly would have less knowledge about the adverse effects of PPI than the population using gastroenterologist-prescribed PPIs. This concern is magnified by the observation that patients taking OTC PPIs are likely more prone to excessive medication use than patients receiving a prescription from a gastroenterologist, because of either suboptimal dose timing or inappropriate indication [38]. Some patients have demonstrated a willingness to reduce unnecessary PPI use on their own, and accordingly, this group may derive an even greater benefit from PPI risk communication than patients with prescription use [20,39,40]. There is a concern about the source of patient information regarding adverse effects associated with PPIs, citing the potential for news, internet, and social media sources to disseminate information of variable quality and without peer review [31,32]. These concerns highlight the need for more provider-driven education regarding PPI risk communication. Such initiatives may also improve adherence among patients who have indications for a PPI but worry about risks that may be incompletely described or exaggerated. Future study should evaluate the potential for educational interventions, including written materials and provider counselling, to improve PPI risk communication. Subsequent study should also consider the impact of educational interventions on changes in dosing behavior and rates of inappropriate use. As most of our study participants were not aware of adverse effects associated with PPIs, we were not able to draw meaningful conclusions about the impact of this awareness on adherence and other behaviors. Secondly, our sample included only patients who were prescribed PPIs by a healthcare provider. It is likely that awareness of reported adverse effects among patients with OTC PPI use is even lower than among patients with prescription use, given the higher rates of inappropriate use observed among the former group [38].

This study highlights the major need for effective provider-driven patient education to enhance awareness of potential risks and reduce inappropriate long-term PPI use, especially in communities with biopsychosocial barriers. As mentioned above, at the time of submission of this manuscript a study was published which showed an increased risk of COVID-19 test positivity among patients taking PPIs [12]. This observation adds to the concern for potentially inappropriate PPI use, especially in the light of the fact that our state is among the top states in the country for number of COVID-19 infections. This is further compounded by the socioeconomic attributes of our community as it has been reported that the impact of COVID-19 is disproportionately higher in medically underserved communities [41]. There is an increased concern for the possibility of low awareness of adverse effects of medications resulting in their overuse. This underscores the overall need for patient education to enhance awareness of potential risks and reduce inappropriate use.

As the next step in improving the quality of the care that our patients receive, we plan to make the outcomes of this study known to the physicians in our healthcare system. We also plan to evaluate the efficacy of multiple interventions for increasing patient awareness of risks associated with long-term PPI use. We will evaluate a simple provider-driven discussion of adverse effects associated with PPIs, as well as the FDA-approved written Medication Guide. The Medication Guide is a brief, patient-oriented section at the end of the manufacturer’s label that includes descriptions of side effects that are associated with the drug. A follow up of continued patient adherence will also be suggested as part of best practices. We will also look into the possibility of making short waiting room patient education videos using TV software that allows one to upload, and mix-in personal videos. These videos will be created both for the inappropriate use of (i) medications in general with PPIs as one example of the medications for broad applicability and (ii) specifically for PPIs alone. It is possible that provider-driven verbal discussion, patient education videos, distribution of the Medication Guide during outpatient visits, or a combination of these interventions will enhance patient awareness of adverse effects associated with PPI use and reduce rates of inappropriate use in our patient community.

## 5. Conclusions

A vast majority of our study participants were not aware of the adverse effects of PPI. Education level of high school or less was associated with a higher risk of long-term PPI use than completion of at least an undergraduate degree. The significantly low patient awareness about PPI adverse effects in our patient population is particularly worrisome, especially as it is related to their demographic attributes. This may lead to long-term and high-dose PPI use. Our study highlights the need for effective provider-driven education regarding medication risks, especially in the communities with significant health disparities.

## Figures and Tables

**Table 1:** Demographics of participating patients.

Characteristic	Outcome (*n =* 101)
**Sex**	
Male	38 (38%)
Female	63 (62%)
**Age (years)**	
18–30	9 (9%)
31–40	9 (9%)
41–50	13 (13%)
51–60	35 (35%)
61–70	19 (18%)
71–80	15 (15%)
>80	1 (1%)
**Race/ethnicity**	
Caucasian	69 (69%)
African American	21 (20%)
Hispanic	10 (10%)
Other	1 (1%)
**Patient’s level of education**	
Less than High School	14 (14%)
High School	50 (49%)
Undergraduate	24 (24%)
Graduate	10 (10%)
Post-Graduate	3 (3%)

Values are presented as *n* (%).

**Table 2:** PPI use characteristics.

Variable	Outcome (*n =* 101)
**PPI prescribing physician**	
Primary Physician	25 (25%)
Gastroenterologist	70 (69%)
Ear, Nose, and Throat specialist	2 (2%)
Other	4 (4%)
**Indications for PPI therapy**	
Gastroesophageal reflux disease (GERD)	65 (64%)
Peptic ulcer disease	5 (5%)
Barrett’s esophagus	14 (14%)
NSAID ulcer prophylaxis	2 (2%)
Dyspepsia	10 (10%)
Other	5 (5%)
**Prescribed PPIs**	
Omeprazole	52 (52%)
Pantoprazole	28 (28%)
Esomeprazole	15 (15%)
Lansoprazole	3 (3%)
Dexlansoprazole	2 (2%)
Rabeprazole	1 (1%)
**Patient knowledge of PPI prescribed dose**	
Yes	58 (57%)
No	43 (43%)
**Duration of PPI therapy**	
<1 year	36 (35%)
1–3 years	22 (22%)
3–5 years	22 (22%)
5–7 years	7 (7%)
7–10 years	4 (4%)
>10 years	10 (10%)
**Patient self-reported adherence**	
Yes	79 (78%)
No	22 (22%)
**Patient awareness of PPI adverse effects**	
Yes	4 (4%)
No	97 (96%)

Values are presented as *n* (%).

**Table 3:** Education level of PPI users.

	<HS (*n =* 14)	HS (*n =* 50)	UG (*n =* 24)	GD (*n =* 13)	≤HS (*n =* 64)	>HS (*n =* 37)	≤HS vs. >HS
Knowledge of dose	8 (57%)	28 (56%)	13 (54%)	9 (69%)	36 (56%)	22 (59%)	*p =* 0.85
Long-term use (≥1 year)	9 (64%)	37 (74%)	9 (37%)	10 (77%)	46 (72%)	19 (51%)	*p =* 0.05
Adherence	13 (93%)	37 (74%)	20 (83%)	9 (69%)	50 (78%)	29 (78%)	*p =* 1.00
Awareness of adverse effects	1 (7%)	1 (2%)	2 (8%)	0 (0%)	2 (3%)	2 (5%)	*p =* 0.62

Values are presented as n (%). *p* ≤ 0.05 was considered statistically significant. <HS *=* Less than high school; HS *=* High school degree; UG *=* Undergraduate degree; GD *=* Graduate/postgraduate degree; ≤HS =  High school degree or less; >HS *=* More than high school degree.

**Table 4:** Race/Ethnicity of PPI users.

	AfA (*n =* 21)	AsA (*n =* 1)	Cau (*n =* 69)	His (*n =* 10)	AfA and His (*n =* 31)	Cau vs. AfA and His
Knowledge of dose	8 (38%)	1 (100%)	48 (70%)	1 (10%)	9 (29%)	*p* < 0.01
Long-term use (≥1 year)	16 (76%)	0 (0%)	42 (61%)	7 (70%)	23 (74%)	*p =* 0.26
Adherence	15 (71%)	0 (0%)	58 (84%)	6 (60%)	21 (68%)	*p =* 0.11
Awareness of adverse effects	0 (0%)	0 (0%)	4 (6%)	0 (0%)	0 (0%)	*p =* 0.31

Values are presented as *n* (%). *p* ≤ 0.05 was considered statistically significant. AfA *=* African American; AsA *=* Asian American; Cau *=* Caucasian; His *=* Hispanic.

**Table 5:** Gender of PPI users.

	Male (*n =* 38)	Female (*n =* 63)	
Knowledge of dose	23 (61%)	35 (56%)	*p =* 0.68
Long-term use (≥1 year)	28 (74%)	37 (59%)	*p =* 0.14
Adherence	28 (74%)	51 (81%)	*p =* 0.46
Awareness of adverse effects	1 (3%)	3 (5%)	*p =* 1.00

Values are presented as *n* (%). *p* ≤ 0.05 was considered statistically significant.

## Supplementary Materials

The following are available online at https://www.mdpi.com/2227-9032/8/4/499/s1.
