# Supplementary material for: Patient Awareness of Reported Adverse Effects Associated with Proton Pump Inhibitors in a Medically Underserved Community
Source: Healthcare (Basel). 2020 Nov 19;8(4):499. doi: 10.3390/healthcare8040499 (PMC7712036; doi:10.3390/healthcare8040499)
Supplement: Supplementary file 1 [file healthcare-08-00499-s001.pdf]

Subject ID Number:

Survey: For patients who are taking PPIs or have taken them in the past year. <u>Please check/circle your choices.</u>

**Age group (years)**	18-30	31-40	41-50	51-60	61-70	71-80	Above 80	
**Gender**	Male	Female	Other					
**Educational level**	Less than high school	High school	Undergraduate	Graduate	Post-Graduate			
**Ethnicity/Race**	African-American	Asian-American	Caucasian	Non-White Hispanic	Other			
**Doctor who prescribed PPI**	Primary Doctor	Gastroenterology (Stomach) Doctor	Ear, Nose, and Throat Doctor	Pulmonologist (Lung Doctor)	Other			
**Which PPI do/did you take?**	Do not know name	Omeprazole (Prilosec)	Pantoprazole (Protonix)	Esomeprazole (Nexium)	Lansoprazole (Prevacid)	Dexlansoprazole (Dexilant)	Rabeprazole (AcipHex)	Other
**Do you know your dose of PPI?**	Yes. Dose:	No						
**How long you were or are on PPI?**	Less than one year	1-3 years	More than 3 to up to 5 years	More than 5 up to 7 years	More than 7 to up to 10 years	More than 10 years		
**How often did your doctor recommend to take PPI?**	Daily	Only when I have symptoms						
**How often do/did you take PPI?**	Less than one time per week	One or two times per week	3-6 times per week	One time everyday	Two times everyday	More than two times everyday		
**Reason to take PPI <u>(choose all that apply)</u>**	Heartburn/Gastro-esophageal reflux disease(GERD)	Stomach or Intestinal Ulcer	Barrett's Esophagus (abnormal lining of esophagus)	I also take drugs like Ibuprofen, Motrin, Advil, Aleve *etc*)	Other	I don't know		
**If you recently stopped your PPI, please indicate your reason for stopping <u>(choose all that apply)</u>**	My doctor stopped prescribing the medication.	It was not working.	I did not know why I was taking the medication.	I was worried that there may be side effects.	I could not afford the medication.	I switched to a different acid medicine. Name of medicine:	Other. List:	
**Are you aware of side effects of PPIs?**	Yes	No						

If you answered no, then this survey is now over. Please read the debriefing document that discusses the known and unknown side effects of PPIs in more detail. **<u>If you answered yes to the previous question, then please continue the survey.</u>**

**Choose all specific side effects of PPIs of which you are aware**	Pneumonia	*Clostridium dificile* infection	Renal (kidney) disease	Dementia	Other. List:			
**How did you hear about PPI side effects?**	News. Source:	Internet. Website:	Medication booklet	Friends/Family	Primary doctor	GI (stomach) doctor	Ear, Nose, and Throat Doctor	Pulmonologist (Lung Doctor)
**Because of side effects, have you changed the dose, frequency, or stopped PPI?**	Yes	No						

If you answered no, then this survey is now over. **<u>If you answered yes to the previous question, then please continue the survey.</u>**

**Have you stopped the medicine completely?**	Yes	No						
**Do you take it less often?**	Yes	No						
**Did you switch to lower dose?**	Yes	No						
**If you stopped completely, did you switch to a different medication?**	Yes. Name of medication:	No						
**Did you discuss any of these changes with your doctor?**	Yes	No						

Thank you for taking the survey.
